# Transposable elements create distinct genomic niches for effector evolution among *Magnaporthe oryzae* lineages

**DOI:** 10.1186/s12915-025-02385-7

**Published:** 2025-09-26

**Authors:** Ana Margarida Sampaio, Daniel Croll

**Affiliations:** Laboratory of Evolutionary Genetics, Institute of Biology, University of Neuchâtel, Neuchâtel, 2000 Switzerland

**Keywords:** *Magnaporthe oryzae*, *AVR* effectors, Transposable elements, Rice blast, Wheat blast

## Abstract

**Background:**

Plant-pathogen interactions are characterized by evolutionary arms races. At the molecular level, fungal effectors can target important plant functions, while plants evolve to improve effector recognition. Rapid evolution in genes encoding effectors can be facilitated by transposable elements (TEs). In *Magnaporthe oryzae*, the causal agent of blast disease in several cereals and grasses, TEs play important roles in chromosomal evolution as well as the gain or loss of effector genes in host specialized lineages. However, a global understanding of TE dynamics driving effector evolution at population scale and across lineages is lacking.

**Results:**

Here, we focus on 16 *AVR* effector loci assessed across a global sampling of 11 reference genomes and 447 newly generated draft genome assemblies from publicly available short-read sequencing data across all major *M. oryzae* lineages and outgroups. We classified each effector based on evidence for duplication, deletion and translocation processes among lineages. Next, we determined *AVR* gain and loss dynamics across lineages allowing for a broad categorization of effector dynamics. Each *AVR* was integrated in a distinct genomic niche determined by the TE activity profile contributing to the diversification at the locus. We quantified TE contributions to effector niches and found that TE identity helped diversify *AVR* loci. We used the large genomic dataset to recapitulate the evolution of the rice blast *AVR1-CO39* locus.

**Conclusions:**

Taken together, our work demonstrates how TE dynamics are an integral component of *M. oryzae* effector evolution, likely facilitating escape from host recognition. In-depth tracking of effector loci is a valuable tool to predict the durability of host resistance.

**Supplementary Information:**

The online version contains supplementary material available at 10.1186/s12915-025-02385-7.

## Background

Plant-pathogen interactions are characterized by continuous evolutionary arms races, where hosts adapt to resist infection while pathogens evolve to overcome host defenses [1]. Effector-receptor dynamics are crucial for this process, where pathogens deploy effectors (*i.e.* avirulence factors, *AVR*) to manipulate host immunity [2, 3], while host receptors are responsible to detect them and trigger defense responses [4, 5]. Genes encoding such effectors are among the most rapidly evolving genes in pathogen genomes, driven by high mutation rates and strong selection pressure [6], enabling pathogens to evade host resistance and posing a challenge for effective disease control. Effector genes are often encoded in subtelomeric chromosomal regions, which are repeat-rich regions and evolve more rapidly than regions encoding housekeeping genes [7, 8]. These genomic regions are also frequently associated with transposable element (TE) activity, which can disrupt coding sequences or promote regulatory changes [9–14]. Moreover, these chromosomal regions are also exhibiting a higher propensity for stochastic epigenetic regulation [15–17], and chromosomal rearrangements resulting in rapid effector gene evolution [18–20].

Plant pathogens capable of infecting both wild host plants and cultivated crops are of particular concern given their propensity to switch to new hosts [21]. *Magnaporthe oryzae* can infect over 50 wild and cultivated grass species including major cereal crops such as rice (*Oryza sativa*) and wheat (*Triticum aestivum*) [22]. Despite the wide host range, *M. oryzae* genotypes are grouping into host-specialized forms and recognized as different pathotypes [23–25]. The most studied pathotypes include *M. oryzae* Oryza (MoO), *M. oryzae* Triticum (MoT), and *M. oryzae* Lolium (MoL), causing blast disease on rice, wheat and ryegrass, respectively. The origin of new pathotypes was triggered by host jumps including the emergence of wheat and rice blast disease [26, 27]. Wheat blast emerged from a host jump from ryegrass (*Lolium* spp.) to Brazilian wheat cultivars lacking the *RWT3* gene, which is the resistance gene expressing the receptor recognizing the PWT3 effector. While both *PWT3* and *PWT4* effector genes are found in the Lolium pathotype, cultivation of *rwt3* wheat cultivars (lacking the ability to recognize PWT3) allowed the emergence of a new Triticum pathotype isolates carrying the PWT3 effector but losing PWT4, an effector that would be recognized due to the presence of its complementary resistance gene (*RWT4*) in wheat cultivars. Subsequent loss-of-function mutations arose due to the nearby cultivation of wheat cultivars carrying *RWT3*, along with the spread of pathogens to common wheat varieties [26]. These findings support the hypothesis that host specialization was mainly driven by genetic changes at effector gene loci including gain or loss of effector functions [28].

Numerous *M. oryzae AVRs* were cloned and characterized for their interaction with plant resistance factors [29]. Among these, AVR-ACE1 is involved in the production of secondary metabolites and activates the rice resistance factor Pi33 [30, 31]. AVR-Piz-t is recognized by Piz-t and suppresses pathogen-associated molecular pattern (PAMP)-triggered immunity [32, 33]. PWL effectors are primarily encoded by MoO and are rapidly evolving, small, glycine-rich secreted proteins [34, 35]. Several AVRs (*i.e.,* AVR-Pi54, Pib, Pik and Pia) are classified as *Magnaporthe* AVRs and ToxB-like (MAX) effectors, sharing a conserved structural fold despite low sequence similarity and being recognized by different R proteins [36, 37]. Beyond functional differences in their encoded proteins, *AVR* genes exhibit a high degree of genetic instability and are often localized in telomeric regions [38, 39]. Such high rates of sequence changes are likely increasing their adaptative potential to evade host recognition. Sequence diversification occurred mostly through simple point mutations as reported for *AVR-Pita* and *AVR-Pik* [40–42], or sequence rearrangements causing segmental deletion of coding sequences as for *AVR-Pita* and *AVR-Pib* [43, 44] associated with gains in virulence. There is also evidence for horizontal transfer of *PWT4* from *M. pennisetigena* to an Avena isolate from Brazil [45]. There is strong evidence that TEs impacted *AVR* loci and facilitate rearrangements. TEs facilitated virulence gains of MoO through loss-of-function mutations such as the insertion of a Mg-SINE into the *AvrPi9* coding sequence [46] or gains of virulence linked to *AVR-Pita* and *AVR-Pib* due to a Pot3 TE insertion [11, 47]. TEs were also likely facilitating the translocations observed for several *AVR* genes including *AVR-Pita* [9]. The loss of telomeric ends resulting in the elimination of *AVR-Pita* and *AVR-Pii* [9, 48] were likely also favored by the repetitive nature of subtelomeric regions. Overall, the rapid evolution of *M. oryzae AVRs* to evade host recognition is likely facilitated by TE dynamics. TEs have specifically expanded in MoO compared to MoT and MoL [49, 50]. Furthermore, TE insertions also mediated the divergence of *M. oryzae* populations infecting different rice subspecies [49, 50]. However, a population genomics perspective on TE impacts on effectors leveraging the vast available genomic datasets on *M. oryzae* lineages is lacking.

Here, we used extensive genomic datasets covering all major *M. oryzae* host-associated pathotypes to recapitulate insertion dynamics near 16 *AVRs* and included *M. grisea* and *M. pennisetigena* as outgroups. We used reference-quality genomes to recapitulate chromosomal rearrangements affecting *AVR* loci across the *M. oryzae* pathotypes MoO, MoT, and MoL. We expanded *AVR* loci investigations at the population-scale by generating draft assemblies from available short-read sequencing datasets in order to assess *AVR* gain/loss patterns in conjunction with TE insertion dynamics*.*

## Results

### Samples distribution, genome assembling and reference genomes

**Fig. 1:**
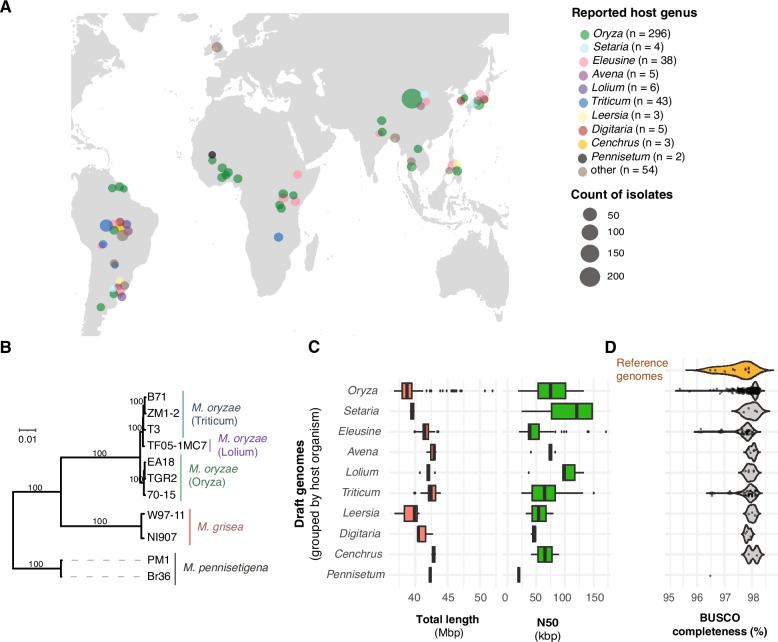
Global panel of analyzed *Magnaporthe* spp. genomes. **A** Geographical distribution of the *Magnaporthe* spp. isolates. The color identifies the reported host genus, and the size defines the number of samples from the same location. **B** Phylogenomic tree of reference-quality genomes. The host genus is reported in parentheses. Bootstrap confidence values > 80% are displayed. **C** *De novo* draft assembly genome size for genomes assembled from short-read sequencing data. Genomes are grouped by reported host genus. **D** Analyses of genome completeness based on BUSCO completeness percentages for reference-quality genomes (orange on top) and *de novo* draft assembled genomes

To unravel *M. oryzae AVR* evolution, we assembled a collection of genomic datasets for 458 *Magnaporthe* spp. isolates collected across continents and diverse hosts (Fig. 1A) [51–54]. Most isolates were collected in Asia (59%) and predominantly infecting *O. sativa* (65%) (Fig. 1A), reflecting the high incidence of rice blast disease in this region. Isolates infecting cereals such as *Triticum* sp. as well as grasses such as *Lolium* sp. and *Eleusine* sp. across continents were also included (Fig. 1A). From the 458 analyzed isolates, 448 belonged to *M. oryzae* (Additional file 2: Table S1). Genomic data included 441 short read sequencing datasets and 7 reference-quality genomes from MoO, MoL and MoT (Fig. 1B; Additional file 2: Table S2). Ten outgroup genomes from two distinct *Magnaporthe* species (*M. grisea* and *M. pennisetigena*) were also included in the analysis of which four genomes were of reference quality (Additional file 2: Table S2). The selected outgroup *Magnaporthe* species *M. grisea* and *M. pennisetigena* clustered separately from each other and separated well from *M. oryzae* in the phylogenomic tree analysis (Fig. 1B). To complement the available reference genomes, we either accessed or assembled draft genomes for 447 additional isolates. Assembly genome sizes ranged from 39.1 Mbp in isolates infecting *Leersia* spp. and 42.9 Mbp in isolates infecting *Cenchrus* sp*.* (Fig. 1C). Assembled genomes for this study showed acceptable contiguity for the purpose of analyzing coding regions with the N50 (Length of the shortest contig for which longer Length contigs cover at least 50% of the assembly) averaging between 19,433 bp in isolates infecting *Pennisetum* sp*.* and 103,607 bp in isolates infecting *Setaria* sp. (Fig. 1C). Assembly genome sizes were not meaningfully correlated with the assembly contiguity (*i.e.* N50), which supports the notion that the totality of the genome is reasonably well covered despite variation in assembly quality (Additional file 1: Fig. S1). The reference-quality genomes showed BUSCO completeness scores ranging from 96.5% in PM1 (*M. pennisetigena*) to 97.9% in *M. oryzae* isolates infecting *Oryza* spp. (Fig. 1D). All draft genome assemblies similarly exhibited > 95% BUSCO completeness and are hence comparable to the reference genomes in terms of gene content. A small number of *Oryza*-infecting isolates showed slightly lower BUSCO completeness scores compared to the reference genomes, yet the assembly genome sizes were similar.

### Rearrangements of *AVRs *among *M. oryzae* reference genomes

**Fig. 2:**
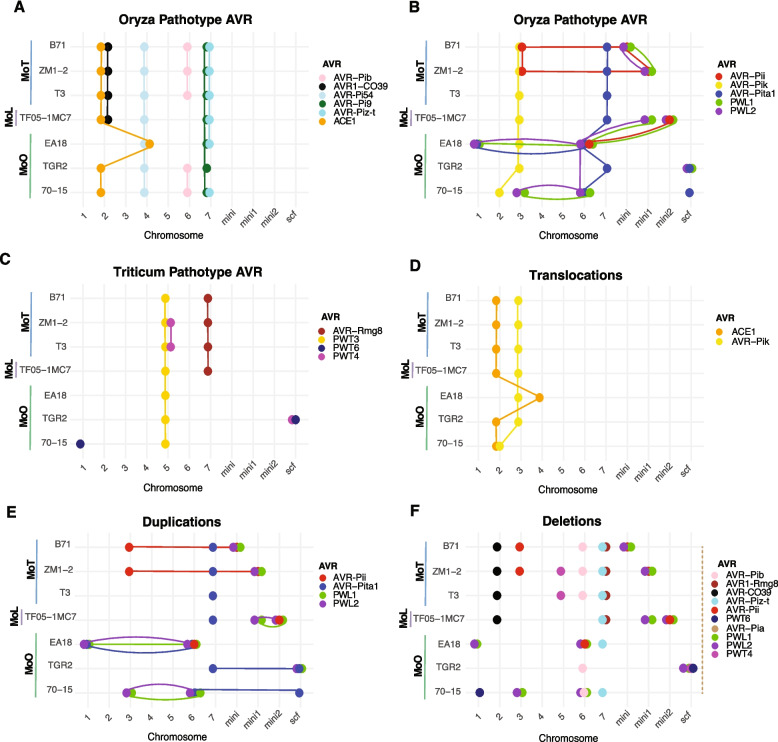
Overview of *AVR* effector synteny among *M. oryzae* reference genomes from MoO, MoL and MoT. Chromosomal localizations of *AVR*s are reported for (**A**, **B**) MoO and (**C**) MoT. Overview of *AVRs* showing evidence for (**D**) translocation among chromosomes, as well as (**E**) duplication and (**F**) deletion events. Lines indicate different types of rearrangements, with their type defined by the plot context. Colored lines connect *AVR* localizations between reference genomes or chromosomes of the same genome (*i.e*. duplications)

Some *M. oryzae AVR* underwent chromosomal translocations. To comprehensively track *AVR* localization among isolates, we focused on 16 cloned and characterized *AVR* effectors (Table 1) in seven reference *M. oryzae* genomes from MoO, MoL and MoT. We use the term “*AVR* pathotype” to identify in which host–pathogen interaction the *AVR* was first reported. However, this does not exclude the possibility that additional pathotypes or hosts share the *AVR* or receptor, respectively. We expected MoO effectors to be shared among most MoO reference genomes. However, from the 12 MoO *AVR* effectors (Table 1), only seven were shared among all MoO reference genomes and only four were found in all *M. oryzae* reference genomes (Fig. 2A, B). These four *AVRs* include *AVR-Pi9* and *AVR-Pi54* being at conserved chromosomal locations within pathotypes, while *ACE1* and *AVR-Pik* showed translocations among MoO isolates (Fig. 2D). *AVR-Pita1*, *PWL1* and *PWL2* were present in all MoO reference genomes but have undergone duplication events (Fig. 2E). We also identified deletions of two MoO *AVRs* (*AVR-Piz-t* and *AVR-Pib*) in at least one MoO reference genome (Fig. 2F). *AVR-Pii* was absent in two MoO reference genomes (Fig. 2F) but exhibited a duplication in two MoT reference genomes (Fig. 2E). *AVR-Pii* was the only MoO effector that does not exhibit a duplication in a reference genome for its associated pathotype but in other *M. oryzae* pathotype reference genomes. *AVR1-CO39* and *AVR-Pia* suffered the most dramatic loss, being absent in all MoO reference genomes (Fig. 2F). *AVR1-CO39* is shared among all MoL and MoT reference genomes though (Fig. 2F). MoT effectors showed similar loss patterns to MoO effectors. Two MoT effectors were shared among the MoT reference genomes (Fig. 2C). *PWT3* is shared among all *M. oryzae* reference genomes and located at a conserved position on chromosome 5 (Fig. 2C). *PWT4* is absent in most *M. oryzae* reference genomes except MoT. *PWT6* exhibited the most pronounced pattern of effector loss with the *AVR* being retained in only two MoO reference genomes (Fig. 2F). Overall, *AVR* localizations are highly dynamic among the seven reference genomes with contributions by translocations, deletions and duplication events.

**Table 1:** Overview of *Magnaporthe oryzae AVR* effectors analyzed in this study. The *AVR* pathotype identifies the *M. oryzae* pathotype in which the effector was first characterized

*AVR* gene	Corresponding resistance gene	*AVR* pathotype ^1^	Sequence NCBI identifier	References
*AVR-Pi9*	*Pi9*	MoO	MW288376.1	[46]
*AVR-Pi54*	*Pi54, Pi54th, Pi54of*	MoO	KY441415.1	[55]
*ACE1*	*Pi33*	MoO	AJ704622.1	[30]
*AVR-Piz-t*	*Piz-t*	MoO	LC175951.1	[32]
*PWL2*	*MLA3*	MoO	MN072512.1	[56, 57]
*AVR-Pib*	*Pib*	MoO	KM887844.1	[44]
*AVR-Pik*	*Pik*	MoO	AB498876.1	[58]
*AVR-Pita1*	*Ptr*	MoO	FJ842897.1	[59]
*AVR-Pii*	*Pii*	MoO	LC175996.1	[58]
*AVR-Pia*	*Pia* (RGA4/RGA5)	MoO	AB498873.1	[58, 60]
*PWL1*	Unknown	MoO	MT669814.1	[34]
*AVR1-CO39*	*Pi-CO39* (RGA4/RGA5)	MoO	AF463528.1	[60, 61]
*AVR-Rmg8*	*Rmg8*	MoT	LC223814.1	[62]
*PWT3*	*Rmg6 (Rwt3)*	MoT	LC202652.1	[26]
*PWT4*	*Rmg1 (Rwt4)*	MoT	LC202656.1	[26]
*PWT6*	*Rmg9 (Rwt6)*	MoT	LC574008.1^2^	[63]

^1^MoO represents the Oryza pathotype, and MoT the Triticum pathotype

^2^Sequence from MoE (Eleusine pathotype)

### Effector gain and loss dynamics across global *M. oryzae* lineages

**Fig. 3:**
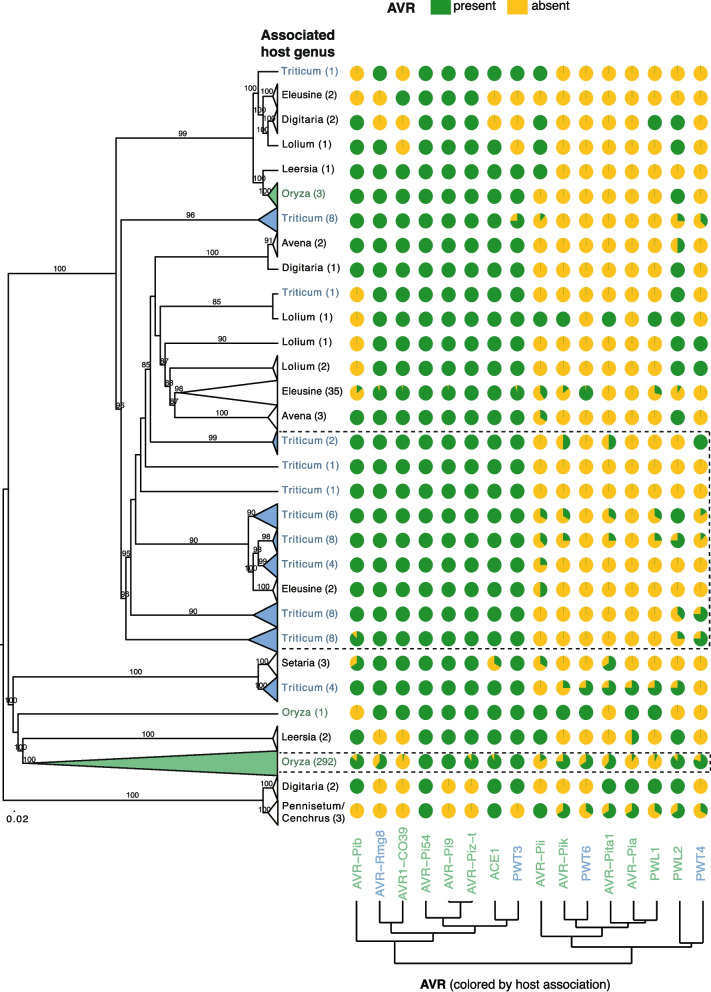
Effectors gain and loss dynamics across *Magnaporthe* spp. Maximum Likelihood phylogenomic tree of 405 *Magnaporthe* genomes from the most frequently sampled pathotypes based on 300 protein sequence alignments of single-copy genes. Bootstrap confidence values > 80% are displayed. Numbers in parentheses indicate the number of genomes. Pie charts indicate the proportion of isolates carrying specific effectors per clade. *AVRs* are organized based on hierarchical clustering of presence/absence patterns. *AVRs* colored in green belong to the MoO *AVR* pathotype and in blue belong to the MoT *AVR* pathotype. A full-sized tree showing *AVR* presence/absence for each isolate is available from Zenodo (https://doi.org/10.5281/zenodo.15875042)

To comprehensively map the evolution trajectory of *AVRs* across *M. oryzae* Lineages, we searched for effector homologs across a dataset of 458 M*. oryzae* genomes (Additional file 2: Table S3). Isolates infecting *Cenchrus* and *Pennisetum* sp. were used as outgroups to clarify gains and loss patterns, since no other reference isolate contains all the investigated *AVRs*. Relationships among isolates were assessed based on a phylogenomic tree including 300 single-copy orthologs focusing only on the most frequently sampled host-associated lineages to reduce complexity. The phylogenetic grouping is consistent with previous studies of *M. oryzae* lineage diversification (Fig. 3). Isolates belonging to the *Avena*, *Lolium* and *Triticum* pathotypes clustered together. Among those, isolates collected from *Triticum* sp. were the most dispersed across the tree, corroborating the high genetic diversity reported for MoT pathotype isolates [64]. We verified consistency of phylogenetic placements for 24 isolates overlapping with a previous phylogenomic analysis [54]. Three isolates reported as collected on *Oryza* sp. leaves (A-PHL-64, ARG-60 and ARG-61) [65] did not cluster with the remaining MoO isolates. Furthermore, four MoT isolates were not clustering as closely with MoL and MoA (*M. oryzae* Avena) as expected but rather with MoS (*M. oryzae* Setaria) (Fig. 3).

Although various genomic rearrangements were observed among reference genomes (Fig. 2), the analysis of the extended *M. oryzae* panel focused only on deletions given the limitations in contiguity of short-read assembled genomes. To categorize effector gain and loss dynamics, we assessed the frequency of effectors according to their reported host (Table 1). Most *AVRs* were present in at least one of the outgroups infecting *Cenchrus* and *Pennisetum* sp., respectively, suggesting that most *M. oryzae* effectors were present in the common ancestor to all extant *M. oryzae* lineages. Exceptions include *AVR-Pi9*, *AVR-Piz-t*, *AVR1-CO39* and *AVR-Rmg8* being absent in the outgroups. *AVRs* were mainly clustered into two groups: *AVRs* with largely stable frequencies among lineages and *AVRs* largely lost in most *M. oryzae* pathotypes, including *AVRs* largely lost in the pathotype reportedly linked to the *AVR* function (Fig. 3). MoO and MoT effectors were found among both main groups (Fig. 3). *AVR-Pi9*, *AVR-Pi54*, *ACE1* and *AVR-Piz-t* are the most well conserved across the *M. oryzae* phylogeny (Fig. 3). The near fixation of the *AVRs* suggests that recognition by the host is not widely distributed among host varieties or that the *AVR* serves an additional function.

### Transposable element colonization near *M. oryzae* effectors

**Fig. 4:**
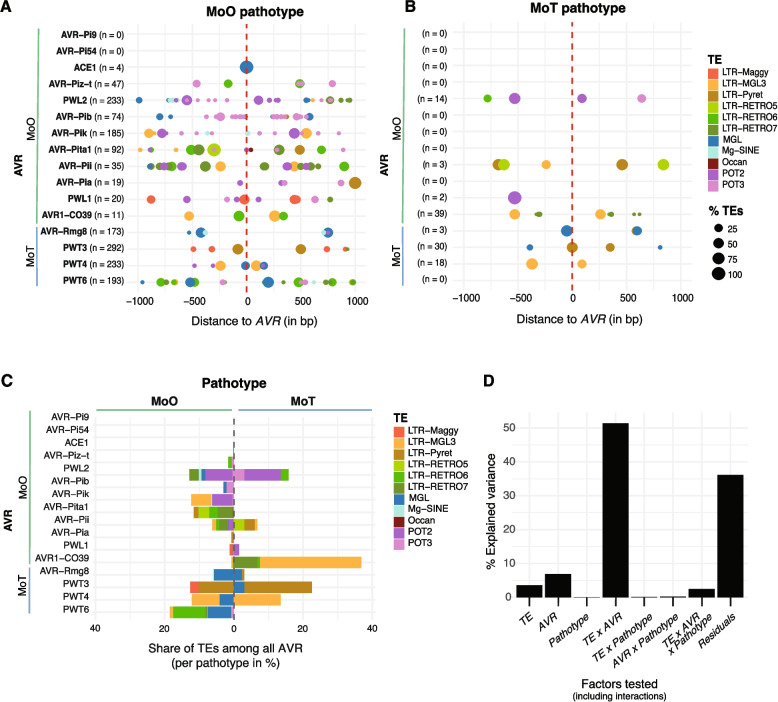
Transposable element (TE) dynamics and diversification near *M. oryzae AVR*. **A** Presence of TEs in a ± 1000 bp window surrounding *AVR* loci in Oryza pathotype (MoO) isolates. The circle color reflects TE identity and classification. The circle size indicates the percentage of isolates carrying the respective *AVR* having a specific TE present. 100% refers to all isolates with the *AVR* sharing a specific TE at a specific position. Counts (*n*) indicate the number of isolates exhibiting at least one TE for a given *AVR*. Negative bp distance values represent *AVR* upstream regions. **B** Triticum pathotype (MoT) isolates. **C** Relative abundance of TEs among *AVR* loci shown separately for MoO and MoT isolates. Percentage calculations consider only isolates carrying the specific *AVR*. **D** Analyses of variance (ANOVA) of factors potentially explaining variation in TE abundance among *AVRs*. The percentage of variance explained by the different models based on factorial combinations for TE, *AVR* and pathotype (for which the *AVR* was first described) and their interactions

The high frequency of rearrangements at *AVR* loci is consistent with high repetitive DNA content nearby. TE insertions are known to facilitate the creation of structural variation. Hence, we investigated patterns of TE dynamics surrounding *AVRs* and potential links to effector presence/absence variation. For this, we used only isolates clustering together with others from the same pathotype in Fig. 3. We annotated assembled contigs encoding the different *AVRs* for the presence of TE sequences considering a window of ± 1000 bp of the effector coding sequence (Additional file 1: Fig. S2; Additional file 2: Table S4). Here we found that the most frequent and conserved effectors (*AVR-Pi9*, *AVR-Pi54*, *ACE1* and *AVR-Piz-t*) loci were devoid or nearly devoid of TEs in proximity in both MoO and MoT isolates (Fig. 4A and B). This suggests that the conservation of *AVR* effectors is facilitated by suppressed TE activity nearby. On the contrary, *AVR1-CO39*, an effector lost nearly entirely in MoO but remaining at high frequency in MoT isolates, showed the highest percentage of TEs among MoT isolates (Fig. 4C). Hence, the high rate of TE insertions nearby could have facilitated the loss of the effector. Interestingly, the frequency of TE families is associated with the *AVR* identity. The two DNA transposons *POT2* and *POT3* are at high frequency near *PWT2*, *AVR-Pib* and *AVR-Pik* loci (Fig. 4C). Retrotransposons were also abundant near specific *AVR*s. *LTR-RETRO5* and *LTR-RETRO7* were commonly found near the MoO effectors *AVR-Pita1*, *AVR-Pii*, and *AVR1-CO39*, while *LTR-RETRO6* was more frequent near the MoT effector *PWT6*. Both *LTR-RETRO7* and* 6* show high sequence similarity with *Inago1* and *2* retrotransposons respectively, which have been shown to be flank several *M. oryzae AVRs* [9, 66]*.* The *LTR-MGL3* retrotransposon showed a high frequency near *AVR1-CO39* and *PWT4*. Notably, *LTR-Pyret* was exclusively detected near *PWT3* in both MoO and MoT isolates. Additionally, the *MGL-LTR* retrotransposon was most frequent near effectors first characterized in MoT (Fig. 4C). The distance between TEs and the nearby effector gene varied. Effectors in MoO had typically a higher distance to TEs than effectors in MoT isolates suggesting that sequence rearrangements could have affected the spacing between TEs and effectors (Fig. 4A and B).

Given the heterogeneity in TE occupancy near *AVRs* across pathotypes, we sought to formally assess what factors explain best the variability in TE content. Using a multi-factorial ANOVA, we found that the TE identity (*i.e.* classification), the identity of the *AVR* and the pathotype explained a significant portion of the variance in TE occupancy (Fig. 4D; Additional file 2: Table S5). The largest proportion of variance (51%) in TE occupancy was explained by the interaction of *AVR* and TE identity. This suggests that *AVR* loci may have co-evolved with distinct TE families, reinforcing the idea that certain TEs have affinity for specific chromosomal regions.

### *AVR1-CO39* locus dynamics

**Fig. 5:**
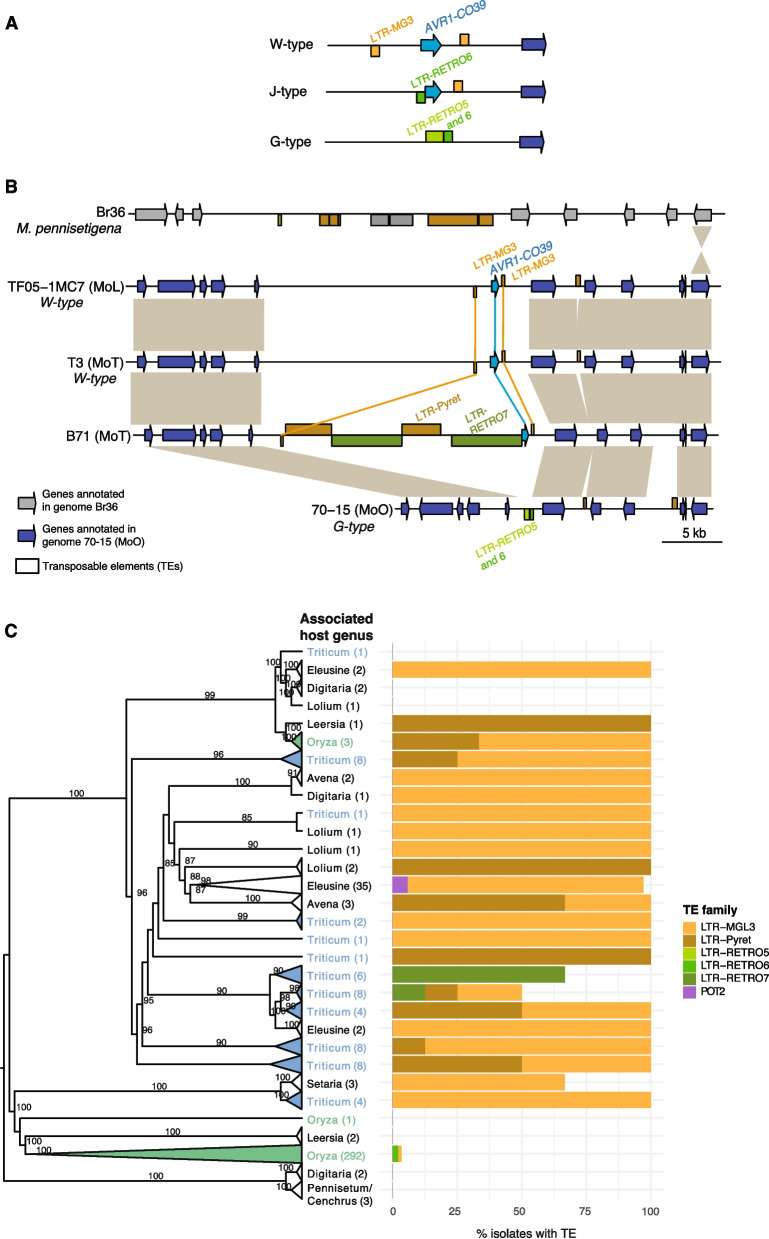
Chromosomal synteny analyses of the *AVR1-CO39* locus. **A** Sketch of *AVR1-CO39* locus types as reported in the literature [69]. **B** Synteny plot of the *AVR1-CO39* region on chromosome 2 of MoO, MoL and MoT reference genomes and *M. pennisetigena*. Grey blocks between chromosomes indicate homologous regions. **C** Comparison of TE types upstream of *AVR1-CO39* among *Magnaporthe* lineages*.* The phylogenomic tree is based on 300 single-copy orthologs. Bootstrap confidence values > 80% are displayed

*AVR1-CO39* was the only effector at low frequency in isolates of the pathotype (MoO) in which the effector function was originally described and at high frequency in all the other pathotypes (Fig. 3). *AVR1-CO39* was previously characterized for a sequence rearrangement at the origin of the host switch to rice [67–69]. The two variants were described as the G- and J-type in MoO lacking a functional *AVR* and an alternative W-type associated with an intact *AVR1-CO39* in isolates infecting weeping love grass [69]. The most frequent type in MoO (G-type) is characterized by a complete loss of the coding sequence through a deletion and TE replacement. The J-type consists of a loss-of-function version caused by a repetitive element called REP1 [69]. REP1 corresponds to LTR-RETRO6 in more recent TE annotations (Fig. 5A). We analyzed the evolution of the *AVR1-CO39* locus organization across pathotypes by inspecting first the reference genomes. We used gene models annotated in the MoO genome 70–15 to obtain gene annotations in the additional *M. oryzae* reference genomes included in the comparison (Fig. 5B). The W-type was represented by the MoL and MoT reference genomes (Fig. 5B). The W-type carries genes adjacent to *AVR1-CO39*, which are absent in 70–15 (empty chromosomal region of the W-type; Fig. 5B). We identified no locus synteny with the outgroup reference genome (Br36) (Fig. 5B). As previously reported for the MoO isolate Guy11 [69], the 70–15 MoO reference genome shows a substantial contraction of the region adjacent to *AVR1-CO39* compared to the MoL and MoT genomes (Fig. 5B).

The contracted region in 70–15 exhibits different TEs compared to the W*-*type, with *LTR-RETRO5* and *LTR-RETRO6* occupying the contracted region. The *LTR-RETRO5* has been previously identified in the G-type and the adjacent element was called *REP1*, which we identified here as *LTR-RETRO6*. The MoT isolate B71 exhibits yet distinct pattern with the *AVR1-CO39* upstream region fully occupied by TEs (Fig. 5B). Two of these TEs were intact *LTR-RETRO7*, suggesting a recent insertion. One of the *LTR-RETRO7* insertion resulted in the truncation of *AVR1-CO39* in B71, differing in size from the J-type rearrangement. However, both retained ORF3, which encodes the *AVR1-CO39* domain interacting with the corresponding resistance gene [70]. The Zambia wheat blast outbreak isolate ZM1-2 (MoT) was reported to share a similar *AVR1-CO39* locus structure as B71 [52]. This highlights that the *AVR1-CO39* loss-of-function is not restricted to MoO isolates but is also occurring independently in MoT isolates through a different TE insertion. Since the different *AVR1-CO39* types are characterized by distinct TE content upstream of the coding sequence, we expanded our investigations to all isolates of the collection carrying the effector. We find that the *LTR-MGL3*, characteristic of the W-type rearrangement, was the most frequent among *Magnaporthe* lineages (Fig. 5C). In contrast, the J-type was rare and found in only 10 MoO isolates (Fig. 5C). The *LTR-RETRO7,* not characteristic of any of the previously described *AVR1-CO39* locus types (Fig. 5A), was found in the reference genome B71 and a small number of additional MoT isolates (Fig. 5C). We identified two further TEs, *LTR-Pyret* and *POT2*, which were also found directly upstream of *AVR1-CO39* in our dataset. Our investigations of the locus indicate that the W-type is likely ancestral and has undergone multiple rearrangements followed by different TE insertions leading ultimately to the loss of the effector (*i.e.* G-type).

## Discussion

*AVR* effectors of *M. oryzae* have undergone rapid evolution to circumvent the matching plant receptors and increase pathogen virulence [71]. Point mutations, insertions and deletions are widely described mechanisms responsible for the loss of avirulence function of *AVR* genes [9, 43, 44, 47]. Abundance of TE sequences near effector regions can act as a mutagen or epigenetic regulator for accelerated effector evolution [72–74]. In *M. oryzae*, TE content correlates with host identity and contributes to genetic differentiation among lineages [49, 50]. Here, we show that effectors evolved well differentiated presence/absence patterns across *Magnaporthe* pathotypes, which at least in part reflects the diversity in recognition mechanisms by the different hosts. Across pathotypes of *M. oryzae*, we show that TE insertion dynamics most likely underpin the observed effector rearrangements. In contrast, conserved *AVRs* show no recent TE activity in the surrounding regions.

Variable *AVR* frequencies among *M. oryzae* host-specific pathotypes may be driven by adaptation to new hosts or the deployment of a new resistance gene. Here, we show that multiple MoO effectors were lost in different *M. oryzae* pathotypes including rice blast. This is consistent with the high rates of gene losses reported for rice-infecting *M. oryzae* compared to *Triticum* or *Avena* sp*.* infecting isolates [28]. How closely the *AVR* frequencies among hosts reflects pathogenicity and recognition capabilities remains to be determined. Effector loss may be sufficient to escape host recognition and therefore contributing to host range expansion. This has been reported for instance for *PWT3*, with its loss coinciding with the widespread deployment of the complementary *R* gene *RWT3* [26]. In contrast, the more recently described *MAX* effectors do not necessarily reflect host specificity [75]. This is consistent with the absence of certain effectors, which may not reflect avoidance of host recognition. Well-conserved effectors across multiple pathotypes and an origin outside of *M. oryzae* may reflect a conserved function in virulence maintained by purifying selection. However, contributions to virulence may still vary among pathotypes, as observed for the *ACE1* effector [31, 76].

TEs represent approximately 10% of the *M. oryzae* genome [72, 77] and can be inserted in or around *AVR* genes altering their virulence spectrum through transcriptional silencing, loss-of-function or loss of avirulence [11, 44, 47, 78, 79]. TE-mediated disruptions can result in the permanent loss of *AVR* genes, being a possible explanation for the observed deletion patterns. In *AVR-Pib*, a POT3 transposon insertion mediates effector loss-of-function in Philippine MoO isolates [80]. We found a striking association of the TE *POT3* in the neighboring regions of *AVR-Pib* in MoO, suggesting that the TE plays a functional role. TE insertions can also promote the emergence of new virulent effector variants, as observed for the MGL retrotransposon insertion into the *ACE1* gene [81] and POT3 insertion in *AVR-Pita1* [11, 82] and *AVR-Piz-t* [32] ensuring the maintenance of such effectors. In our collection, all the four MoO isolates exhibiting TEs in the *ACE1* surrounding regions exhibited this same TE overlapping with the *ACE1* coding sequence. The remaining 270 MoO isolates were devoid of TEs near the effector. TEs can also facilitate the translocation of *AVR* genes such as *AVR-Pita* [9]. Here, we focused on MoO reference-quality genomes to investigate translocations of *ACE1* and *AVR-Pik*. However, we found no association between these translocations and specific TEs.

*M. oryzae* shows lineage-specific TE activity with LTR-retrotransposons expanded in certain lineages, rather than undergoing a single expansion followed by selective deletion [49]. TEs were often found near genes with presence/absence variation including effectors [83]. Here, we observed that well maintained *AVRs* had almost no TE activity in the surrounding area. This is possibly explained by their chromosomal location. In contrast to most of the *M. oryzae AVR* genes, usually located in telomeric or subtelomeric regions [9, 43, 58, 84, 85], highly conserved *AVRs* could have been selected to occupy euchromatic and repeat-poor regions. *AVR-Pi9*, a widely present effector among *M. oryzae* pathotypes, is located in a genomic region close to the chromosome 7 centromere, which is one of the most stable regions of the genome [46]. Similarly, *AVR-Piz-t* is found at high frequency among pathotypes and located 230 kb from *AVR-Pi9* [32]. Effectors with highly variable presence/absence frequencies among pathotypes include *AVR-Pia*, *AVR-Pii*, *AVR-Pita* and *PWL* and are well-known to be located in unstable chromosomal regions such as subtelomeres prone to rearrangement [34, 35, 43, 58, 86]. Despite the broad spectrum in chromosomal locations, most of the effectors are expressed during the early stages of infection. AVR-Pi9 accumulates in the biotrophic interfacial complex structure and is translocated in the early stages of infection to the host cell [46]. A similar expression and translocation pattern was also observed for less conserved effectors such as AVR-Pia and AVR-Pita [87–89].

TE activity is typically high in telomeric and subtelomeric regions [8, 90]. Here, we show that *AVRs* with highly variable frequencies among pathotypes are indeed surrounded by TEs. The presence of TE can also affect epigenetic regulation through changes in heterochromatic structure [15–17]. These results reinforce the idea that in *M. oryzae* different TE environments impact effector evolution in distinct ways. Our findings also indicate that certain TE superfamilies may have more affinity for specific *AVR*s loci, reflected also in the fact that the TE presence is largely conserved among pathotypes. This suggests that despite differences in TE activity among fungal plant pathogens [91–93], including in *M. oryzae* [49, 50], the effector regions may have been selected for a variety of genomic niche features. The clearest evidence for TE insertions creating transitional states at effector loci was found for *AVR1-CO39*, with TEs likely contributing to the loss in MoO. Future studies will be able to significantly expand on *AVR* locus dynamics by shifting to a comprehensive set of long-read genomes. This will overcome the limitations of draft assemblies resolving only *AVR-*proximate sequence dynamics.

## Conclusions

Overall, we show that *M. oryzae AVR* locus evolution was characterized by parallel and well differentiation dynamics in TEs. Spanning the spectrum of effectors exhibiting rapid changes in frequencies across *M. oryzae* pathotypes to conserved effector loci retained at high frequency and devoid of TEs. This highlights that host-mediated selection plays not only a role in *AVR* frequencies but that the genomic niche of the effectors displays likely similar associated dynamics. The dynamics of TE insertions within plant pathogens as a response to host selection remains understudied. Our work demonstrates though that TE dynamics can be an integral component of genomic niche evolution. In-depth tracking of effector niches will likely augment our ability to predict the durability of host resistance.

## Methods

### *Magnaporthe* genomic datasets and genome assemblies

We performed analyses on a global collection of genomic datasets comprising 458 *Magnaporthe* isolates (Additional file 2: Tables S1 and S2). All genomes were accessed from public databases reported by previous studies [51–54]. The geographic origin of isolates and the host organism information was retrieved from metadata attributes and cross-checked with the associated literature. The isolates were from Asia (*n* = 256), South America (*n* = 96), Africa (*n* = 69), Europe (*n* = 11), North America (*n* = 8), and a small number without reported origin (*n* = 18). Out of the 458 isolates, 448 belonged to *M. oryzae* collected from 18 different host species, including cereals and grasses. We also included as outgroups two isolates of *M. pennisetigena* (collected on *Pennisetum* sp.) and eight isolates of *M. grisea* isolated from *Cenchrus* and *Digitaria* sp. Overall, 447 isolates were sequenced using Illumina paired-end whole-genome sequencing (WGS) (Additional file 2: Table S1). Illumina sequencing data was initially filtered using fastp v0.23.4 with default settings [94] to remove adapter sequence and low-quality reads. De novo draft assemblies were generated using the software SPAdes v3.15.5 [95] with the “careful” method and automated k-mer selection. All genomes were verified to have more than 95% completeness using BUSCO version 5.8.2 [96] searching the Ascomycota orthology database. We used QUAST to calculate assembly metrics [97]. We retained assemblies with N50 (Length of the shortest contig for which longer Length contigs cover at least 50% of the assembly) above 17,034 bp and a total assembly size above 36.72 Mbp. Eleven *Magnaporthe* reference-quality genome assemblies were also included in this study [77, 98–100]. These genomes included seven *M. oryzae* genomes (3 MoO, 3 MoT, 1 MoL) and four outgroup genomes (2 M*. grisea* and 2 M*. pennisetigena*) (Additional file 2: Table S2).

### Phylogenetic analyses

Phylogenetic relationships were assessed separately for the set of *Magnaporthe* reference-quality genomes and for 405 *Magnaporthe* draft genomes associated with the most frequently sampled host species. For the phylogenomic tree, we used single-copy genes predicted by AUGUSTUS v3.5.0 [101] using the pretrained gene prediction database available for the *M. grisea* genome. Predicted protein sequences were used for orthology analyses performed with Orthofinder v2.5.5 [102]. Single-copy orthologs present in at Least 90% of the total number of isolates were kept. For the phylogenetic reconstruction of the large *Magnaporthe* worldwide collection, we retained 300 randomly selected single-copy orthologs to reduce computational load. Selected ortholog protein sequences were aligned as a supermatrix using the AMAS tool v1.0 [103]. Phylogenetic trees were built using RAxML v8 [104] to construct a maximum-likelihood phylogenetic tree with the parameters -m PROTGAMMAAUTO for protein sequences with 1000 bootstrap replicates. We used the *hclust *function in R to perform hierarchical clustering of *AVRs*.

### Identification of effector homologues

We analyzed the 447 draft genomes produced by SPAdes and the 11 reference-quality genomes (including outgroups) to search for homologs of 16 M*. oryzae* effectors. We focused on cloned and well-characterized effectors identified in Oryza or Triticum pathotype isolates (Table 1). For effectors present in more than one pathotype, effector sequences with best hits in the reference-quality genomes were used as query for BLASTn analyses [105]. Hits were filtered for a maximum *e*-value of 10^–5^, followed by individual minimum length filtering for each effector based on visual inspection of alignment length distributions. The ORF finder tool (https://www.ncbi.nlm.nih.gov/orffinder/) was used to refine effector open reading frames detected by BLASTn hits.

### Transposable element annotation and analysis

To detect TE insertions near effectors, all draft assemblies, reference-quality and outgroup genomes were annotated with the TE consensus sequences reported for *Magnaporthe* [50] (available from https://github.com/S-t-ing/mBio-data-availablility/blob/main/Mo.TE_Consensus.fasta). For this, we used RepeatMasker v4.0.9_p2 with “-no_is” and “-nolow” parameters to skip simple repeat and low complexity region annotations. Annotated TEs shorter than 50 bp were filtered out. The search for TEs near effector loci was restricted to a 1000 bp window up- and downstream the effector gene. Only MoO and MoT isolates clearly identified in large clades based on the phylogenetic tree were retained these analyses. Percentage of TE superfamilies per *AVR* in MoO and MoT was calculated per effector based on the total TE number per pathotype. Percentage of TE superfamilies per insertion site (bp) has been calculated per effector based on the total number of isolates containing at least one TE for this *AVR.* We used the *aov* function in R to perform an ANOVA testing the effects of the following factors: TE family, AVR identity, and AVR pathotype (MoO vs. MoT) with the response variable being TE counts near effectors. We assessed how much each factor, and interactions thereof, contributed to the overall variation by using the sum of squares from the ANOVA.

### Analyses of the *AVR1-CO39* locus

To assess the *AVR1-CO39* locus organization in genomes of different pathotypes, we first analyzed reference-quality genomes. Synteny was plotted with genoplotR v0.8.11 [106] for the *Magnaporthe* reference genomes using gene and TE annotation [50]. For *M. oryzae* isolates, we mapped coding sequences from the MoO 70–15 (GCF_000002495.2) gene annotation [77], and for outgroup *M. pennisetigena* we mapped coding sequences from the Br36 (GCA_004337985.1) gene annotation [98]. The two annotations of 70–15 and Br36 provided complementary coverage of major haplotypes found at the locus. AUGUSTUS gene annotations as described above were used to infer coding sequences in non-*MoO* isolates. *Magnaporthe* genomes assembly having *AVR1-CO39* were inspected for TE presence surrounding the effector gene.

## Supplementary Information

Additional file 1: Figures S1-S2. Figure S1: Correlation between total genome assembly size and N50 metrics for de novo assembled genomes. Figure S2: Presence of TEs in a +/- 1000 bp window surrounding AVR loci in Setaria (MoS), Eleusine (MoE), Avena (MoA), Lolium (MoL), Leersia (MoLe), Digitaria (MoD), Cenchrus (MoC), and Pennisetum (MoP) pathotype isolates.

Additional file 2: Tables S1-S5. Table S1: Analyzed Magnaporthe oryzae isolates sequenced by Illumina paired-end whole-genome sequencing. Table S2: Analyzed reference-quality Magnaporthe genomes. Table S3: Blast hit statistics to identify the different AVR genes in the Magnaporthe genomes (draft and reference genomes). Table S4: Transposable elements (TEs) detected near each AVR. Table S5: ANOVA of TE occupancy near AVRs.

## Data Availability

The data analyzed in the frame of this study were retrieved from NCBI repositories as indicated in the Additional File 2. Draft genome assembly data, a Magnaporthe spp. full-size phylogenetic tree with AVR presence/absence screening is available from Zenodo (10.5281/zenodo.15875042) [107].

## Abbreviations

TEs: Transposable elements
AVR: Avirulence effector
Mo: *Magnaporthe oryzae*
MoO: *Magnaporthe oryzae* Oryza
MoT: *Magnaporthe oryzae* Triticum
MoL: *Magnaporthe oryzae* Lolium
MoE: *Magnaporthe oryzae* Eleusine
MoA: *Magnaporthe oryzae* Avena
MoS: *Magnaporthe oryzae* Setaria
PAMP: Pathogen-associated molecular pattern
R proteins/genes: Resistance protein/genes
