# Supplementary material for: Transposable elements create distinct genomic niches for effector evolution among Magnaporthe oryzae lineages
Source: BMC Biol. 2025 Sep 26;23:282. doi: 10.1186/s12915-025-02385-7 (PMC12465478; doi:10.1186/s12915-025-02385-7)
Supplement: Supplementary file 1 — Additional file 1: Figures S1-S2. Figure S1: Correlation between total genome assembly size and N50 metrics for de novo assembled genomes. Figure S2: Presence of TEs in a +/- 1000 bp window surrounding AVR loci in Setaria (MoS), Eleusine (MoE), Avena (MoA), Lolium (MoL), Leersia (MoLe), Digitaria (MoD), Cenchrus (MoC), and Pennisetum (MoP) pathotype isolates. [file 12915_2025_2385_MOESM1_ESM.pdf]

Additional file 1

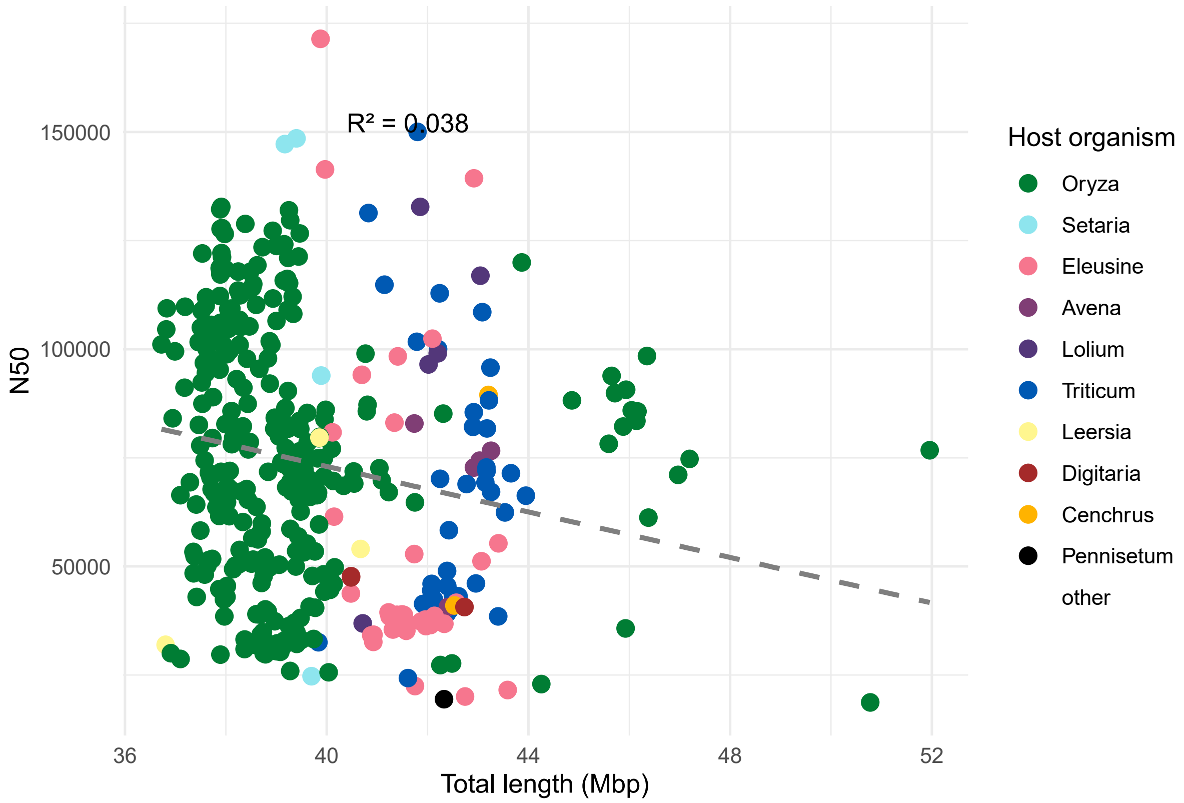

Figure S1: Correlation between total genome assembly size and N50 metrics for *de novo* assembled genomes. Colors identify the reported host for each isolate.

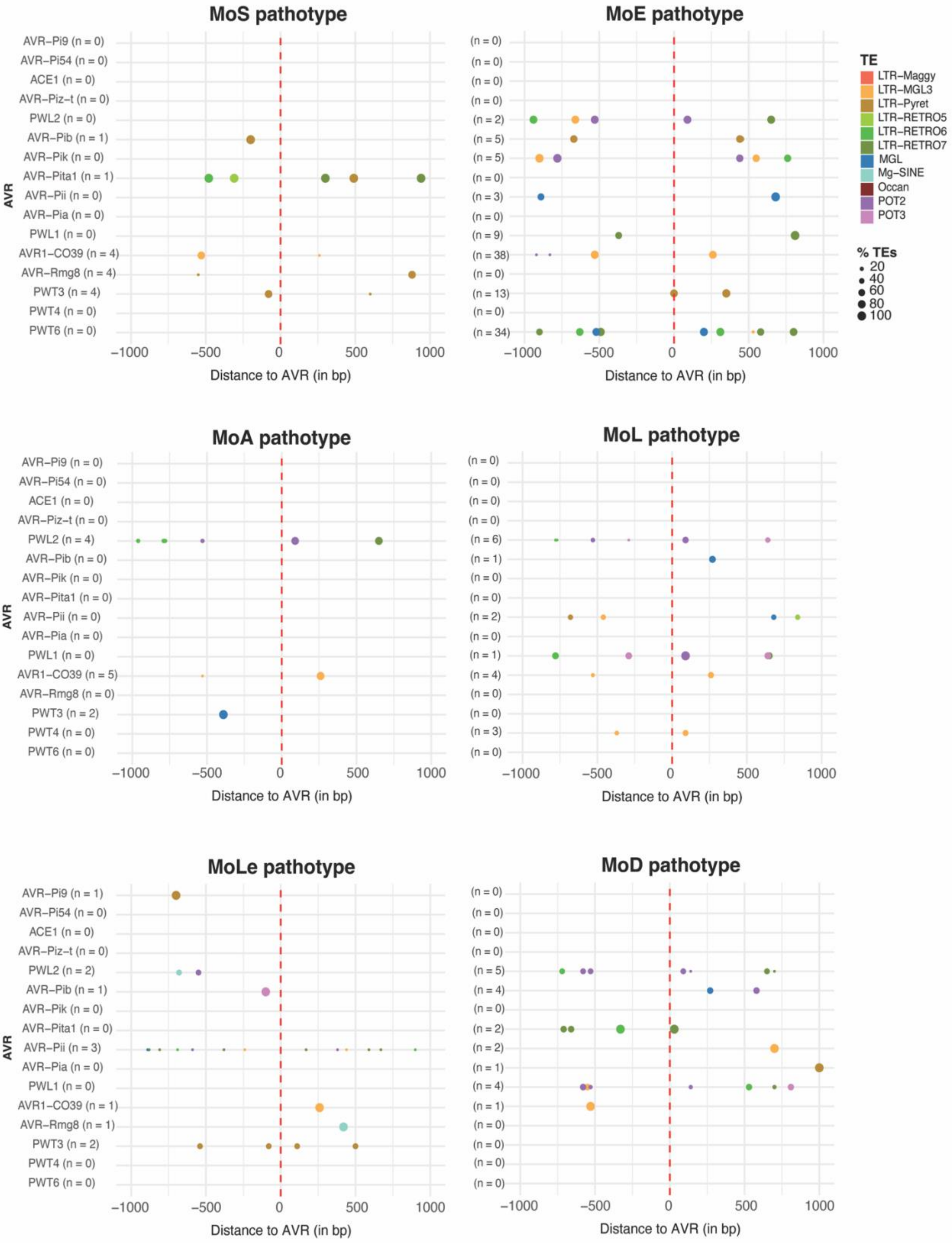

MoS pathotype
MoE pathotype
MoA pathotype
MoL pathotype
MoLe pathotype
MoD pathotype
AVR
AVR–Pi9
AVR–Pi54
ACE1
AVR–Piz–t
PWL2
AVR–Pib
AVR–Pik
AVR–Pita1
AVR–Pii
AVR–Pia
PWL1
AVR1–CO39
AVR–Rmg8
PWT3
PWT4
PWT6
Distance to AVR (in bp)
TE
LTR–Maggy
LTR–MGL3
LTR–Pyret
LTR–RETRO5
LTR–RETRO6
LTR–RETRO7
MGL
Mg–SINE
Occan
POT2
POT3
% TEs
20
40
60
80
100

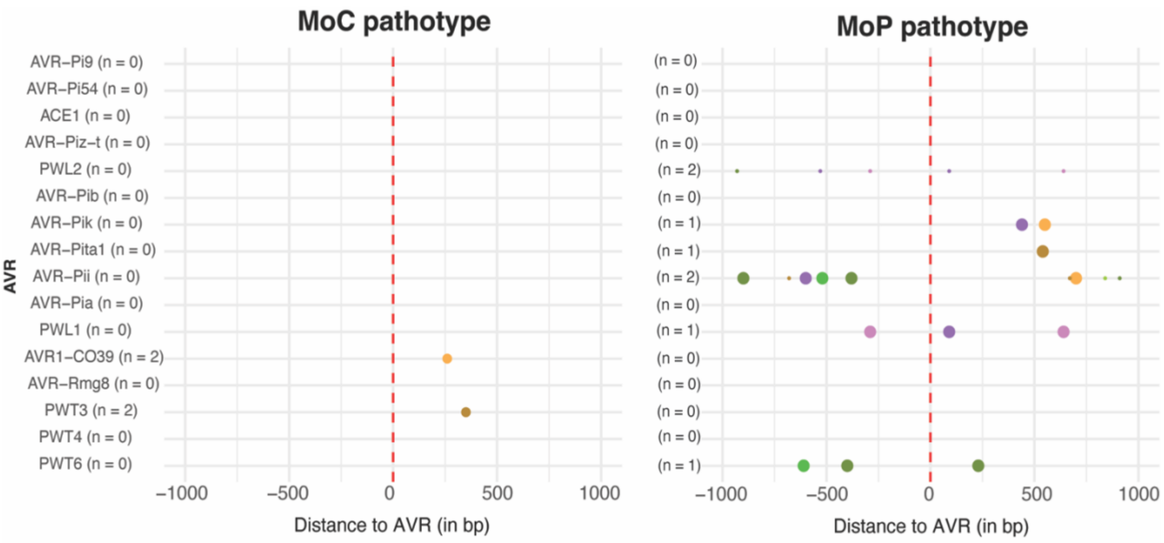

Figure S2: Presence of TEs in a +/- 1000 bp window surrounding *AVR* loci in Setaria (MoS), Eleusine (MoE), Avena (MoA), Lolium (MoL), Leersia (MoLe), Digitaria (MoD), Cenchrus (MoC), and Pennisetum (MoP) pathotype isolates. The circle color reflects TE identity and classification. The circle size indicates the percentage of isolates carrying the respective *AVR* having a specific TE present. 100% refers to all isolates with the *AVR* sharing a specific TE at a specific position. Counts (*n*) indicate the number of isolates exhibiting at least one TE for a given *AVR*. Negative bp distance values represent *AVR* upstream regions.
